# The Philosophy of Extracting Teeth by Electricity, without Pain. The Human Body a Galvanic Battery and Magnetic Telegraph

**Published:** 1859-04

**Authors:** Samuel B. Smith

**Affiliations:** Electrician, New-York.


### ARTICLE VIII.

*The Philosophy of Extracting Teeth by Electricity, without Pain. The Human Body a Galvanic Battery and Magnetic Telegraph.* BY SAMUEL B. SMITH, Electrician, New-York.

THE brain is the positive pole of the human body, or the principal telegraph office. Let us call the brain, New-York, and the jaw, &c. the different stations throughout the body.

In the human body there are two sets of nerves, the nerves of motion and those of sensation.

By the nerves of motion, the mind transmits its mandates from the brain, and moves those parts of the body where motion is required. By the nerves of sensation, pain or pleasure is transmitted to the brain. All this is effected by the vital fluid passing along the nerves, either from or to the brain, as the case may be. Now, as the brain is the principal telegraph office, there is a king who presides there. When he wants anything done at any of the stations of his empire, he transmits his will down the *nerves of motion*, and his will is obeyed; for instance, he wants the hand to touch the foot, and the foot is touched. Then, again, if an enemy attack his empire, for instance, a tooth is being extracted, immediately the pain is transmitted along the nerve to the brain; the king takes cognizance of it, and acts accordingly.

Having now a pretty good knowledge of the use of the nerves, so far as connected with the subject under consideration, we shall examine the characteristics of the different magnetic machines now offered to the public, as anæsthetics or alleviators of pain in the extracting of teeth.

The ordinary electro-magnetic machines give out only a to-and-fro current. The one invented by myself, and patented, is the *only one* which gives out both the to-and-fro current, and a *direct current.*

No to-and-fro current magnetic machine can prevent pain in the extracting of a tooth. None but a direct current can possibly do it. This, it now behooves me to prove.

First, then, a to-and-fro current is a current which runs *backwards and forwards* like the pendulum of a clock. It has no specific polar action, because its poles change polarity every time the current is broken by the vibration of the spring.

The direct current runs *but one way,* like a river running down stream. There are several ways of proving the characteristic differences of these currents. I shall here mention but one, referring the reader, for further proof, to my "medical application of electro-magnetism," pp. 89 and 90. Take, for instance, a solution of silver; into this place a piece of polished brass, suspended on a wire from the negative pole of the direct current of my magnetic machine, and, suspend in the same solution, a piece of silver, say a ten cent coin, on a wire from the positive pole, and, in a few minutes, it will be completely plated with silver. Perform the same experiment with a to-and-fro current machine, and no plating will be effected. (Try this experiment with the ordinary magnetic machines, and the truth will be discovered.)

Now, for the applying these facts to the pulling of teeth without pain:

Here is a direct current magnetic machine. The patient takes the positive pole of the instrument in his hand, the negative pole being in connection with the nerve of the tooth through the forceps, or some other way, or through a spring pressing on the nerve externally. The galvanic and electric current now passes up the arm and through the nerve of the tooth back to the battery. In its course it passes along the nerve of sensation leading to the tooth, and thus prevents the pain of extracting, by preventing the nervous or vital fluid from reaching the brain. This may be illustrated as follows: let the nerve be represented by a leaden tube, at one end of which is a reservoir of water being poured continually through the tube into the tooth. Let this water represent the electric current, which performs the functions of the nervous fluid. Under these circumstances no water could pass the contrary way at the same time. Such, precisely, is the case with the direct electric current; consequently, no pain could reach the brain, as two positive electric currents cannot pass along the same line, in different directions, at the same time. Neither can the nervous fluid pass from the tooth to the brain, so long as the electric current is passing along the same nerve in an opposite direction.

But with the to-and-fro current there is an interval between the vibrations of the spring, or the break in the current, when the nervous force rushes to the brain and causes pain: the pendulum goes to-and-fro from the tooth to the brain, and back again. The monarch on his throne feels this pain, and shrieks. Not so with the *direct* current. He knows nothing of what is going on, except his eyes or his ears tell him, and then he don't believe them.

